# Supplementary figures and images for: Identification of two glycosyltransferases required for synthesis of membrane glycolipids in Clostridioides difficile
Source: mBio. 2025 Feb 18;16(3):e03512-24. doi: 10.1128/mbio.03512-24 (PMC11898633; doi:10.1128/mbio.03512-24)

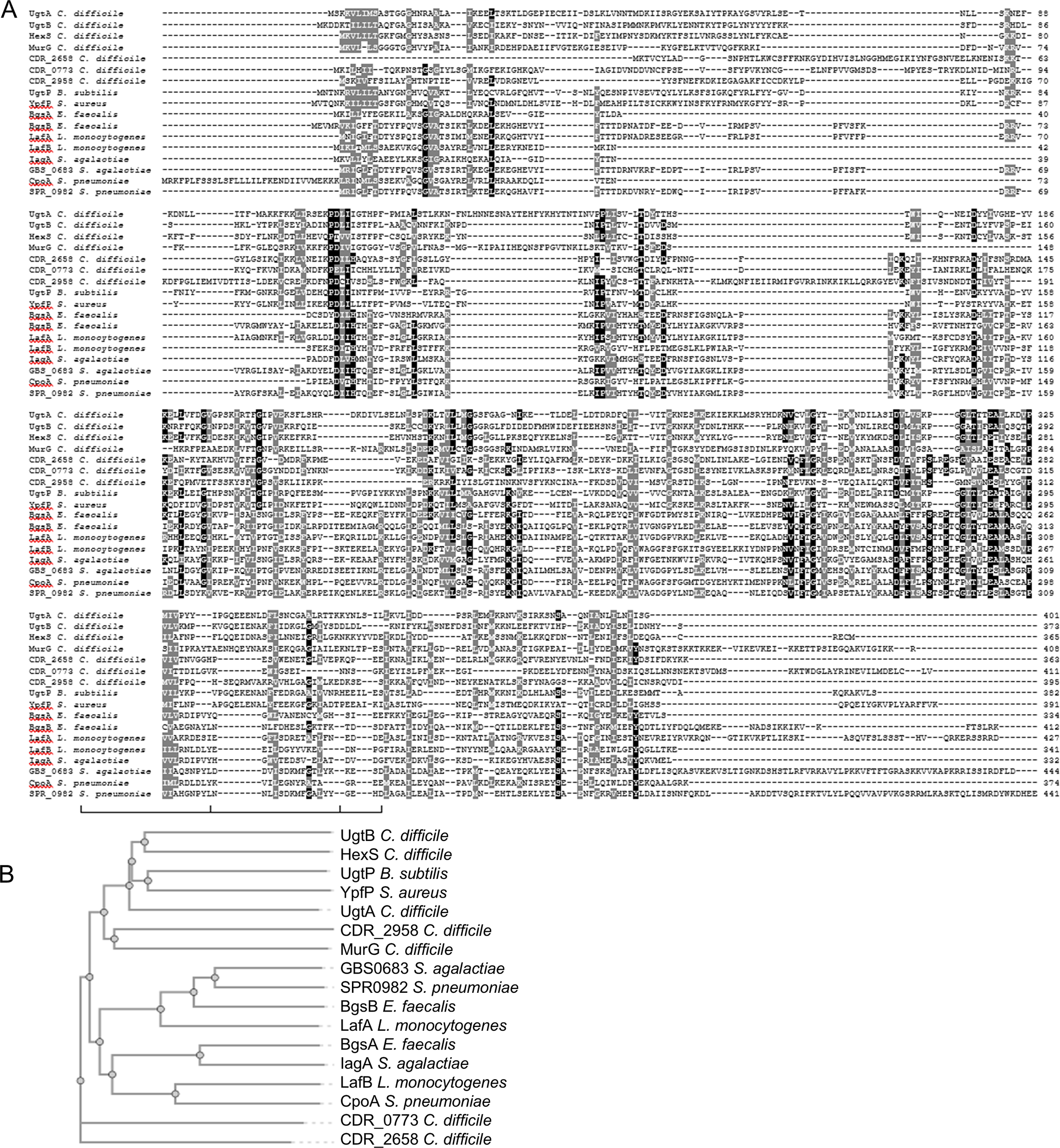

Supplement: Fig. S1 — Amino acid alignment of glycosyltransferases. [file mbio.03512-24-s0001.tif]

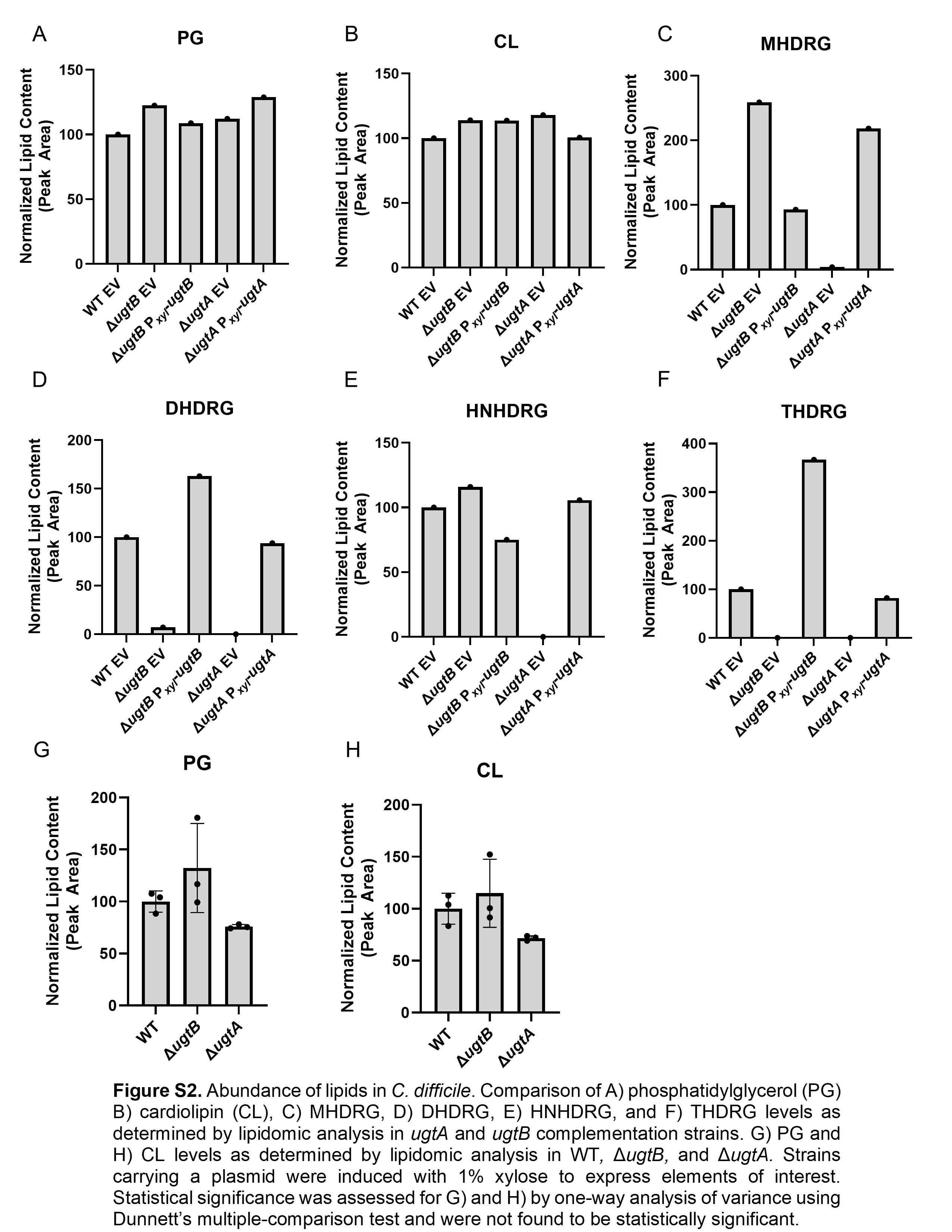

Supplement: Fig. S2 — Abundance of lipids in C. difficile. [file mbio.03512-24-s0002.tiff]

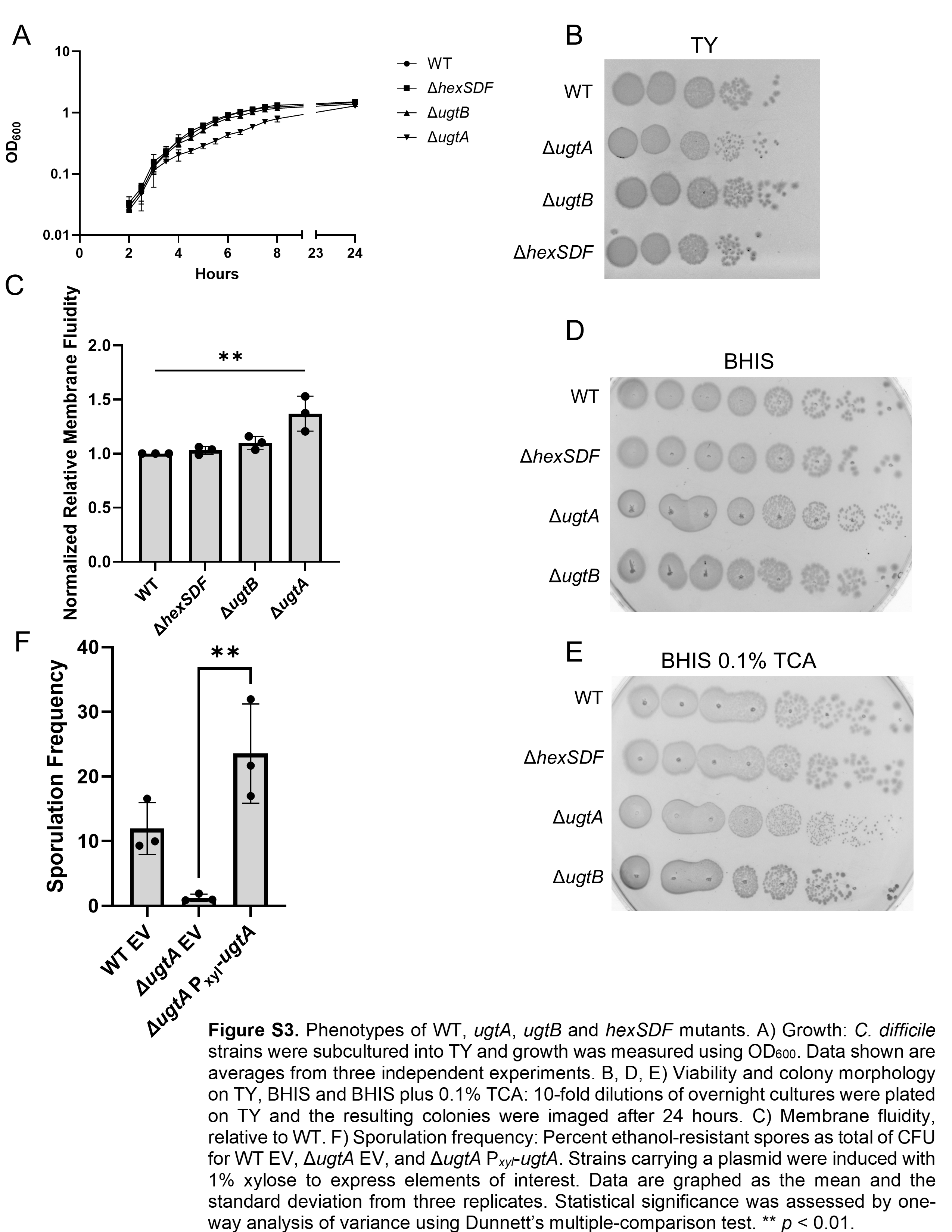

Supplement: Fig. S3 — Phenotypes of WT and ugtA, ugtB, and hexSDF mutants. [file mbio.03512-24-s0003.tiff]

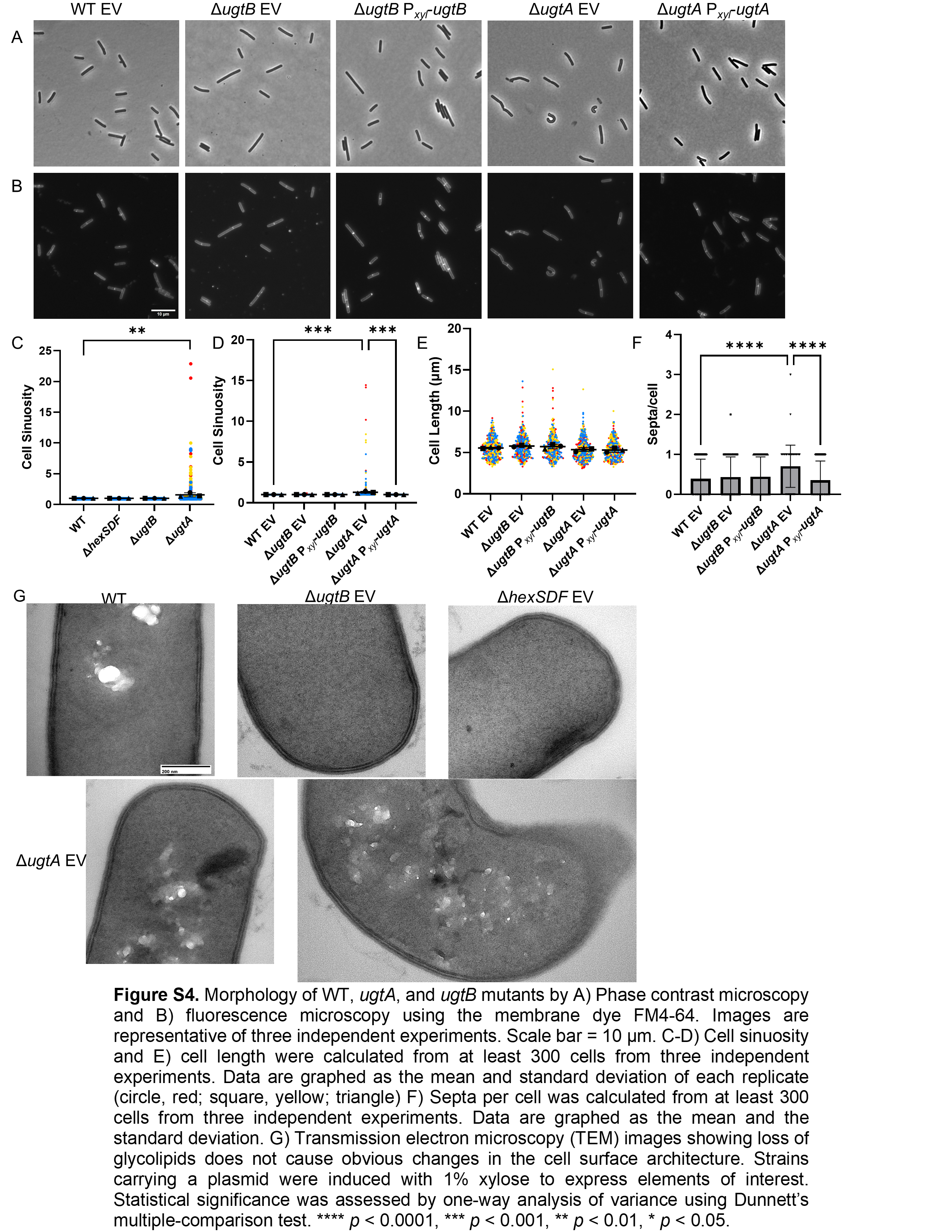

Supplement: Fig. S4 — Morphology of WT and ugtA and ugtB mutants. [file mbio.03512-24-s0004.tif]

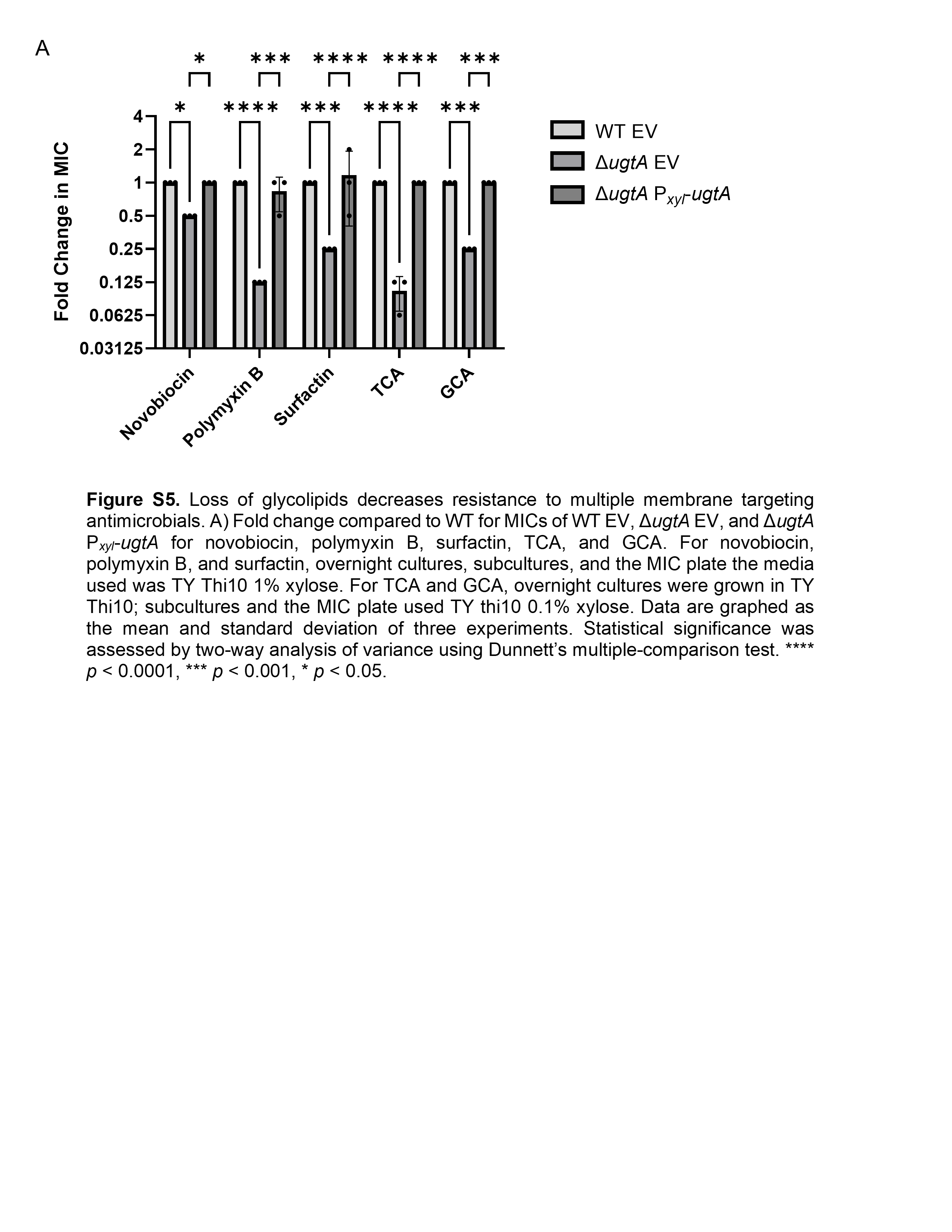

Supplement: Fig. S5 — Loss of glycolipids decreases resistance to multiple membrane-targeting antimicrobials. [file mbio.03512-24-s0005.tiff]
