# Supplementary material for: Identification of two glycosyltransferases required for synthesis of membrane glycolipids in Clostridioides difficile
Source: mBio. 2025 Feb 18;16(3):e03512-24. doi: 10.1128/mbio.03512-24 (PMC11898633; doi:10.1128/mbio.03512-24)
Supplement: Table S1 — BLAST results of diacylglycerol glucosyltransferases. [file mbio.03512-24-s0006.pdf]

Table S1 BLAST Results of diacylglycerol glucosyltransferases

Protein	Product Name	Score (Bits)	E Value	Query Protein	Query Organism	PFAM Family	PFAM Description
CDR20291_0008	putative glycosyl transferase	164	5.00E-48	UgtP	*B. subtilis*	PF06925.16;PF04101.21	MGDG_synth;Glyco_tran_28_C
CDR20291_1186	putative cell wall biosynthesis protein	146	3.00E-41	UgtP	*B. subtilis*	PF06925.16;PF04101.21	MGDG_synth;Glyco_tran_28_C
HexS	putative monogalactosyldiacylglycerol synthase	129	4.00E-35	UgtP	*B. subtilis*	PF06925.16;PF04101.21	MGDG_synth;Glyco_tran_28_C
CDR20291_0773	putative glycosyl transferase	58.5	1.00E-10	LafB	*L. monocytogenes*	PF13439.11;PF00534.25	Glyco_transf_4;Glycos_transf_1
CDR20291_2658	putative capsular polysaccharide biosynthesis glycosyl transferase	48.5	4.00E-07	SPR_0982	*S. pneumoniae*	PF13477.11;PF00534.25	Glyco_trans_4_2;Glycos_transf_1
MurG	UDP-N-acetylglucosamine--N-acetylmuramyl-(penta peptide) pyrophosphoryl-undecaprenol N-acetylglucosamine transferase	47.4	8.00E-07	UgtP	*B. subtilis*	PF03033.25;PF04101.21	Glyco_transf_28;Glyco_tran_28_C
CDR20291_2958	putative glycosyltransferase	43.1	2.00E-05	UgtP	*B. subtilis*	PF00201.23	UDPGT
