# Supplementary material for: Identification of two glycosyltransferases required for synthesis of membrane glycolipids in Clostridioides difficile
Source: mBio. 2025 Feb 18;16(3):e03512-24. doi: 10.1128/mbio.03512-24 (PMC11898633; doi:10.1128/mbio.03512-24)
Supplement: Table S2 — MICs. [file mbio.03512-24-s0007.pdf]

Table S2 MIC

**MIC**	WT	Δ*hexSDF*	Δ*ugtB*	Δ*ugtA*
Ampicillin (µg/ml)	1.33 ± 0.58	0.8 ± 0	1 ± 0	0.75 ± 0.43
Bacitracin (µg/ml)	256 ± 0 ^	64 ± 0 ^	256 ± 0 ^	128 ± 0 ^
Cefoxitin (µg/ml)	256 ± 0 ^	ND	ND	256 ± 0 ^
Daptomycin (µg/ml)	4 ± 0	**0.833 ± 0.29 ******	4 ± 0	4 ± 0
Fosfomycin (µg/ml)	16 ± 0 ^	ND	16 ± 0 ^	32 ± 0 ^
Meropenem (µg/ml)	3.33 ± 1.15	ND	ND	3.33 ± 1.15
Nisin (µg/ml)	180 ± 0	120 ± 51.96	**90 ± 0 ****	**90 ± 0 ****
Vancomycin (µg/ml)	1 ± 0 ^	ND	0.5 ± 0 ^	1 ± 0 ^
Lauric Acid (mM)	0.25 ± 0 ^	0.25 ± 0 ^	0.25 ± 0 ^	0.25 ± 0 ^
Linoleic Acid (mM)	>32	>32	>32	>32
Lysozyme (mg/ml)	8 ± 0	6.67 ± 2.31	**4 ± 0 ***	**3.33 ± 1.15 ****
Novobiocin (µg/ml)	16 ± 0	16 ± 0	16 ± 0	10.67 ± 4.62

ND-not determined
Mean ± standard deviation
* $p < 0.05$
** $p < 0.01$
**** $p < 0.0001$
^Did not calculate statistical significance using one-way analysis of variance as the standard deviation was 0.
