# Supplementary material for: Identification of two glycosyltransferases required for synthesis of membrane glycolipids in Clostridioides difficile
Source: mBio. 2025 Feb 18;16(3):e03512-24. doi: 10.1128/mbio.03512-24 (PMC11898633; doi:10.1128/mbio.03512-24)
Supplement: Table S3 — Plasmids. [file mbio.03512-24-s0008.pdf]

Table S3 Plasmids

Plasmid	Relevant features	Parent vector	Restriction enzymes to digest parent plasmid	Primers used	PCR template	Assembly	Reference
pAP114	P_{xyl}::*mCherryOpt cat*						50
pCE971	P_{xyl}::*ugtB cat*	pAP114	BamHI, SacI	CDEP5693-5694	R20291	ITA	
pBZ139	P_{xyl}::*ugtA cat*	pAP114	BamHI, SacI	CDEP6171-6172	R20291	ITA	
pCE678	P_{xyl}::*Cas9-opt* P_{gdh}::*sgRNA-pgdA-2* homology to delete *pgdA cat*						52
pCE1062	P_{xyl}::*Cas9-opt* P_{gdh}::*sgRNA-pgdA-2* homology to delete *ugtB cat*	pCE678	NotI, XhoI	CDEP6257-6260	R20291	ITA	
pCE1065	P_{xyl}::*Cas9-opt* P_{gdh}::*sgRNA-ugtB cat*	pCE1062	MscI, MluI	CDEP4237, 6271	pCE678	ITA	
pCE1069	P_{xyl}::*Cas9-opt* P_{gdh}::*sgRNA-pgdA-2* homology to delete *ugtA cat*	pCE678	NotI, XhoI	CDEP6290-6293	R20291	ITA	
pCE1071	P_{xyl}::*Cas9-opt* P_{gdh}::*sgRNA-ugtA cat*	pCE1069	MscI, MluI	CDEP4237, 6274	pCE678	ITA	
pCE1088	P_{xyl}::*Cas9-opt* P_{gdh}::*sgRNA-pgdA-2* homology to delete *cdr_0773 cat*	pCE678	NotI, XhoI	CDEP6458-6461	R20291	ITA	
pCE1098	P_{xyl}::*Cas9-opt* P_{gdh}::*sgRNA-cdr_0773 cat*	pCE1088	MscI, MluI	CDEP4237, 6462	pCE678	ITA	
pCE1085	P_{xyl}::*Cas9-opt* P_{gdh}::*sgRNA-pgdA-2* homology to delete *cdr_2958 cat*	pCE678	NotI, XhoI	CDEP6454-6457	R20291	ITA	
pCE1105	P_{xyl}::*Cas9-opt* P_{gdh}::*sgRNA-cdr_2958 cat*	pCE1085	MscI, MluI	CDEP4237, 6469	pCE678	ITA	
pDR111	*amyE*:: P_{IPTG} *amp spec*						
pCE1057	*amyE*:: P_{IPTG}::*ugtB amp spec*	pDR111	HindIII, SphI	CDEP6229-6230	R20291	ITA	
pCE1059	*amyE*:: P_{IPTG}::*ugtA amp spec*	pDR111	HindIII, SphI	CDEP6227-6228	R20291	ITA	
pAC68	*thrC*:: P_{xyl} *amp erm*						
pCE1122	*thrC*::P_{xyl}::*ugtB amp erm*	pAC68	HindIII, BamHI	CDEP6615-6616	R20291	ITA	
pCE1281	*thrC*::P_{xyl}::*ugtA amp erm*	pAC68	HindIII, BamHI	CDEP6628-6629	R20291	ITA	
pCE1062	*thrC*::P_{xyl}::*hexSDF amp erm*	pAC68	HindIII, BamHI	CDEP6215-6216	R20291	ITA	
