# Supplementary material for: Identification of two glycosyltransferases required for synthesis of membrane glycolipids in Clostridioides difficile
Source: mBio. 2025 Feb 18;16(3):e03512-24. doi: 10.1128/mbio.03512-24 (PMC11898633; doi:10.1128/mbio.03512-24)
Supplement: Table S4 — Oligonucleotides. [file mbio.03512-24-s0009.pdf]

Table S4 Oligos

Oligo	Sequence	Relevant Features
CDEP5693	cgatagttatgaagtgagcttaaggaggtggctaaaatgaaagatgataaaacaat	Clone *ugtB* onto pAP114
CDEP5694	gttttattaaaacttataggatcttaactgtaatgattatctattaattctgatatataatcagca	Clone *ugtB* onto pAP114
CDEP6171	cgatagttatgaagtgagcttaaggaggattttattatgagcaaaaaagtattaataatg	Clone *ugtA* onto pAP114
CDEP6172	aaagttttattaaaacttataggatcgggattttatttattttatcaaccaga	Clone *ugtA* onto pAP114
CDEP6257	aaacagctatgaccgcggccccctcatcttcacttattaaaatct	Clone homology for *ugtB* deletion
CDEP6258	tgtttgaaatttttaatagtacaattttagccaatttatcaccactt	Clone homology for *ugtB* deletion
CDEP6259	agtggtgataaattggctaaaattgtactattaaaaatttcaaacataaaatt	Clone homology for *ugtB* deletion
CDEP6260	ttatttttatgctagctcgagaaaaacttagaaatatatgcttcaaaa	Clone homology for *ugtB* deletion
CDEP4237	cttataggatccgcggccgctag	Clone *gRNA* from pCE678
CDEP6271	aattaaactgtaaatggccactaatgctaactaagcctgggttttagagctagaaatagc	*sgRNA-ugtB*
CDEP6290	aaacagctatgaccgcggccgtccgccgctctttaccgaa	Clone homology for *ugtA* deletion
CDEP6291	ccctaatagataatatatatactatagggcctcctaaaatgtagcatagtatttatattg	Clone homology for *ugtA* deletion
CDEP6292	ctatgctacattttaggaggccctatagtatatatattatctattagggattt	Clone homology for *ugtA* deletion
CDEP6293	ttatttttatgctagctcgagaaataataatggcaacaaatcct	Clone homology for *ugtA* deletion
CDEP6274	aattaaactgtaaatggccagtattagtaagcaaacctgggttttagagctagaaatagc	*sgRNA-ugtA*
CDEP6458	aaacagctatgaccgcggccggagattgtgctttgtttattaaa	Clone homology for *cdr0773* deletion
CDEP6459	agttacactttttataatttaaataatttttcacttgcctcctttctaaaa	Clone homology for *cdr0773* deletion
CDEP6460	agaaaggaggcaagtgaaaaaattatttaaattataaaaaagtgtaactatttttaaac	Clone homology for *cdr0773* deletion
CDEP6461	ttatttttatgctagctcgacttgtatctactctatctacacca	Clone homology for *cdr0773* deletion
CDEP6462	aattaaactgtaaatggccaggataaaaacttacttcactgttttagagctagaaatagc	*sgRNA-cdr0773*
CDEP6454	aaacagctatgaccgcggccctgacagcaccgttattaaa	Clone homology for *cdr2958* deletion
CDEP6455	tgcatgtatgtatatatatttttaacaaatctaatttcatacctccttttataattttttaaaatca	Clone homology for *cdr2958* deletion
CDEP6456	ttataaaaggaggtatgaaattagatttgttaaaaatatatatacatacatgcaatttaatcaa	Clone homology for *cdr2958* deletion
CDEP6457	ttatttttatgctagctcgacctcattacttcttaaaagtttattattattt	Clone homology for *cdr2958* deletion
CDEP6469	aattaaactgtaaatggccaattctctgctccgaatgttggttttagagctagaaatagc	*sgRNA-cdr2958*
CDEP6615	gacctgcaggcatgcaagcttaaggaggtggctaaaatgaa	Clone *ugtB* onto pAC68
CDEP6616	aaaactgctgccttcggatcttaactgtaatgattatctattaattctgatat	Clone *ugtB* onto pAC68
CDEP6229	aacaattaagcttaaggaggtggctaaaatgaaagatgataaaac	Clone *ugtB* onto pDR111
CDEP6230	ccaccgaattagcttgcatgttaactgtaatgattatctattaattctgatat	Clone *ugtB* onto pDR111
CDEP6628	gacctgcaggcatgcaagctttaggaggattttattatgagcaaa	Clone *ugtA* onto pAC68
CDEP6629	aaaactgctgccttcggatctcaaccagaaattatatttaaaattaaattagc	Clone *ugtA* onto pAC68
CDEP6227	aacaattaagcttaaggaggattttattatgagcaaaaaagtattaataatgt	Clone *ugtA* onto pDR111
CDEP6228	ccaccgaattagcttgcatggggattttatttattttatcaaccaga	Clone *ugtA* onto pDR111
CDEP6215	ccaccgaattagcttgcatgttatcttacataagcaactttttca	Clone *hexSDF* onto pDR111
CDEP6216	gagcggataacaattaagctgctttaacgaggagggaatc	Clone *hexSDF* onto pDR111
